# Hepatitis B virus X protein impedes the DNA repair via its association with transcription factor, TFIIH

**DOI:** 10.1186/1471-2180-11-48

**Published:** 2011-03-04

**Authors:** Ishtiaq Qadri, Kaneez Fatima, Hany AbdeL-Hafiz

**Affiliations:** 1 NUST Center of Virology and Immunology, National University of Science and Technology, Academic Block, Kashmir Highway, H-12 Islamabad, Pakistan; 2 Department of Medicine, University of Colorado Health Sciences Center at Fitzsimons, Aurora, CO, USA

## Abstract

**Background:**

Hepatitis B virus (HBV) infections play an important role in the development of hepatocellular carcinoma (HCC). HBV X protein (HBx) is a multifunctional protein that can modulate various cellular processes and plays a crucial role in the pathogenesis of HCC. HBx is known to interact with DNA helicase components of TFIIH, a basal transcriptional factor and an integral component of DNA excision repair.

**Results:**

In this study, the functional relevance of this association was further investigated in the context to DNA repair. By site-directed mutagenesis HBx's critical residues for interaction with TFIIH were identified. Similarly, TFIIH mutants lacking ATPase domain and the conserved carboxyl-terminal domain failed to interact with HBx. Yeast and mammalian cells expressing HBx^wt ^conferred hypersensitivity to UV irradiation, which is interpreted as a basic deficiency in nucleotide excision repair. HBx^mut120 ^(Glu to Val) was defective in binding to TFIIH and failed to respond to UV.

**Conclusions:**

We conclude that HBx may act as the promoting factor by inhibiting DNA repair causing DNA damage and accumulation of errors, thereby contributing to HCC development.

## Background

Hepatitis B virus (HBV) infection in humans is a major health problem and is one of the principal causative agents of liver disease. It is estimated that over 500 million individuals are infected with HBV worldwide and 1 million deaths are annually attributed to the effects of HBV infection [1-3]. The virus is associated with both acute and chronic liver disease. Although the sequence of events in the development of hepatocellular carcinoma remains poorly defined, a significant correlation has been made between long-term carriage of the virus and the development of HCC [1]. Modes of HBV infection are generally from mother to infant (vertical) and by sexual routes. The direct or indirect role of HBV in the development of HCC appears complex. First, in the absence of reproducible in vitro HBV propagation system, the pathogenesis steps are poorly understood. Second, there is a lack of evidence for HBV replication in tumor cells that arise in HBV infected patients, despite active replication in surrounding non-tumorous hepatocytes. Furthermore, it has been observed that virtually 100% of woodchucks chronically infected with WHV at birth develop liver cancer and die of HCC [4]. Several mechanisms by which HBV infection could lead to the development of HCC have been proposed. These mechanisms include insertional mutagenesis upon integration, trans-activation of the cellular genes, activation of signaling pathways, inactivation of tumor suppressor proteins, synergy with environmental carcinogenesis and host immune response.

One of the open reading frames of the HBV genome encodes a protein termed HBx. HBx is required for viral infection and has been implicated in virus-mediated liver oncogenesis. The HBx protein has been detected in liver tissue from patients with chronic HBV infection, cirrhosis and hepatoma [5-10]. It is now generally acknowledged that HBx supplied in trans can increase gene expression of a wide variety of viral and cellular promoters and enhancer elements [11,12]. Recent studies have demonstrated that HBx possesses both cytoplasmic and nuclear specific activities. A number of cytoplasmic activities have been attributed to HBx including, the activation of Ras/Raf/mitogen-activated protein (MAP) kinase, MEKK1/Jun kinase, [13] protein kinase C signal transduction pathways [14]. Several putative nuclear targets of HBx have been revealed including, ATF-2/CREB transcriptional factors [15-17], RNA polymerase subunit RPB5 [18,19], tumor suppressor protein p53 [20,21], TATA binding protein (TBP) [22], a putative DNA repair protein UV-DDB [23,24], TFIIH [25,26], and RNA polymerase II [19]. AP-2 and C/EBP have also been implicated as potential targets of HBx [27]. HBx has been shown to stimulate transcription by RNA Polymerase II and III [28]. Further, HBx was shown to induce either p53-mediated [29] or tumor necrosis factor alpha (TNFα)-mediated apoptotic destruction of liver cells [30-32].

The functional role of HBx during the HBV life cycle was defined by transfecting a mutant HBV genome, lacking functional HBx. In this case, a poor production of viral proteins was observed [33]. In woodchucks an essential functional role of HBx in vivo was revealed, by the use of HBx mutant. HBx (-) mutant of woodchuck failed to replicate in their natural host [34]. Although, in woodchucks HBx was shown to be important for establishment of virus infection [34,35], the molecular mechanism of HBx activity and its possible influence on cell proliferation remains obscure.

We have shown that HBx interacts with the XPD/ERCC2 and XPB/ERCC3 components of TFIIH and stimulates the DNA helicase activity of TFIIH [25]. This was further substantiated by Haviv and co-workers [28]. Further, we showed that HBx interacts with single-stranded nucleic acids in vitro [36], the implications of which in DNA repair process remains to be investigated. TFIIH is a multiprotein complex of 10 polypeptides [37]. Apart from being an important factor of basal transcriptional machinery, TFIIH has been clearly shown to be an integral component of the DNA repair pathway [38-41].

In this study we explore the physiological relevance of HBx's association with TFIIH in the context of DNA excision repair. Although, interaction of HBx with a probable cellular repair protein UV-DDB was earlier reported by Lee and co-workers [42], a functional role in DNA repair which may result in lethal or hepatocarcinogenic mutations is not understood. This is also primarily due to the fact that a more defined role of UV-DDB in vitro DNA repair reaction is not established. Aboussekhra and co-workers [43,44] have shown that the addition of UV-DDB during in vitro DNA repair reaction had a very modest effect on the repair synthesis. On the other hand TFIIH has been shown to be an essential component of DNA repair both in vivo and in vitro [43,45,46] Support for the role of HBx in DNA repair comes from experiments with the S. cerevisiae and mammalian cells expressing HBx, which displayed an increased UV hypersensitivity. Because of the high degree of homology between yeast and mammalian NER machinery, we have chosen yeast nuclear extracts to investigate the biochemical role of HBx in NER in vitro. Further, S. cerevisiae offers an elegant genetic background to identify the pathways by which HBx may affect this process. In this context, we used mutant yeast extracts with various genetic mutations to investigate the role of HBx in the NER pathways. Our results are consistent with the hypothesis that HBx impedes the DNA repair process.

## Methods

### Saccharomyces cerevisiae strains and plasmids

The genotype of S. cerevisiae wild type strain 334 is MATα pep4-3 prb1-1122 ura3-52 leu2-3, 112 regI-50 gal1. Two NER defective yeast strains rad 1 and rad51 were employed in this study. The genotype of Rad1 is (α rad1-2 his3Δ1 leu2-3-112 lys 1-1 trp1-289 ura3-52) and rad 51 (α rad51-1 his3Δ1 leu2-3-112 lys 1-1 trp1-289 ura3-52). Plasmids pUC18 and pBR322 were used for repair synthesis assays and were purified as described [47]. Plasmid pSBDR contains sequences encoded by an HP1 to Taq1 fragment derived from HBV adw strain which includes enhancer 1 element followed by X promoter, the HBx coding sequences and the polyA addition site. In addition, pSBDR contains neomycin resistance marker for selection in eukaryotic cells.

### UV survival profile of HBx expressing yeast cells

Yeast cultures of strain 334 containing plasmids, pYES and pYES-X^wt ^and pYES-X^mutant ^(as indicated) were grown in 2 ml of YMIN media (0.17% yeast nitrogen base, 1% succinic acid, 0.6% NaOH and 0.5% Ammonium sulfate) with 2% glucose. Saturated yeast cultures were washed in water and resuspended into 2 ml of sterile water. Then 200 μl of washed cells were added into 2 ml of fresh YMIN media containing 2% glycerol, 2% ethanol and 2% galactose for the induction of HBx and grown with shaking (200 rpm) for 24 h. Various cell dilutions were plated simultaneously onto two sets of YMIN plates containing 2% glycerol, 2% ethanol and 2% galactose. One set of plates was immediately irradiated under a germicidal lamp for various dosages of UV light and another set of control plates was not exposed to UV-irradiation. Plates were then incubated in dark for at least 24 h and shifted to 30°C. Colonies were counted to determine the survival fraction.

### UV survival profile of HBx expressing human liver cells

HBx expression plasmid pSBDR and UV-damaged pRC/CMV were co transfected into Chang liver cells. Plates were incubated in dark for 2 weeks in the presence of 0.4 mg/ml of G-418. The number of G-418 resistant clones per 10^5 ^cells is plotted. Live cells were counted by staining with trypan blue after transfection and prior to G-418 selection.

### Yeast nuclear extracts

Yeast cells were grown at 30°C in 1 liter YPD medium (1% yeast extracts, 2% Bactopeptone, 2% Dextrose) to logarithmic phase. Cells were harvested by centrifugation for 10 min, washed in water, and suspended at 0.1 g/ml in 0.1 M EDTA pH 8.0/10 mM dithiothreitol. After incubation at 30°C with shaking (50 rpm) for 10 min, cells were pelleted by centrifugation as described above and suspended at 1 ml in YPS solution (1% yeast extract, 2% Bactopepetone and 1 M sorbitol) and yeast lytic enzyme (ICN) was added at 150 U/g of cells. Following incubation at 30°C with shaking (50 rpm) for 2 hrs, ice cold YPS solution was added (10 mg/g of cells). Spheroblasts were pelleted by centrifugation as above and washed three times in the same buffer. Phenylmethanesulfonyl flouride was added (0.5 mM) before the final centrifugation. Cells were washed again in 1M sorbitol and suspended at 0.125 g/ml in 5 mM Tris-HCl, (pH7.4) 20 mM KCl, 2 mM EDTA-KOH, (pH 7.4), 0.125 mM sperimidine, 0.05 M sperimine, 18% Ficoll, 1% thiodiglycol and with protease inhibitors. Spheroplasts were lysed in a motor-driven homogenizer with 10 strokes. The lysates were centrifuged in a sorvall SW34 rotor at 10000 rpm for 10 min and then for 5 min at 4°C. The nuclei were harvested by centrifugation at 13000 rpm for 30 min at 4°C. Nuclei were resuspended (0.6 ml/g of nuclei) in 100 mM Tris acetate (pH 7.9), 50 mM Potassium Acetate, 10 mM MgSO_4_, 2 mM EDTA, 3 mM DTT, 20% glycerol and protease inhibitors. Then, a solution of 4M NH_4_SO_4 _neutralized with NaOH was slowly added to 0.9 M, gently stirred and centrifuged in a sorvall SW34 rotor at 12000 rpm for 1 h at 4°C. The supernatant was adjusted to 75% saturation with solid NH_4_SO_4 _and neutralized with NaOH. Precipitates were collected by centrifugation in a sorvall SW34 rotor at 12000 rpm for 15 min at 4°C, resuspended in 1/15th volume of high-speed supernatant in 20 mM Hepes-KOH (pH 7.6), 10 mM MgSO_4_, 5 mM DTT, 10 mM EGTA, 20% glycerol (v/v) and protease inhibitors and dialyzed against the same buffer. Precipitates formed during dialysis were removed by centrifugation and the resulting nuclear extracts were stored at -70°C.

### In vitro DNA repair reaction

The repair reaction contained, 0.3 μg of unirradiated pUC18 and 0.3 μg of UV irradiated pBR322 substrate, 45 mM HEPES-KOH (pH 7.8), 70 mM KCl, 7.4 mM MgCl_2_, 0.9 mM DTT, 0.4 mM EDTA, 2 mM ATP, 20 mM each of dGTP, dCTP, and dTTP, and 8 μM dATP, 2 μCi [α-32]dATP (3000 Ci/mmol), 40 mM phosphocreatine, 2.5 mg creatine phosphokinase (type 1), 3.4% glycerol, 18 mg bovine serum albumin and 100 μg of cell extracts. Reactions were incubated for 6 h at 30°C. Reactions were stopped by the addition of EDTA and then incubated with RNAse, SDS and proteinase K. Plasmids were digested with HindIII and loaded on 1% agarose gel. After overnight electrophoresis, the gel was photographed under near-UV transillumination with Polaroid film and an autoradiograph of the dried gel was obtained.

### Synthesis and purification of an oligonucleotide containing a single 1.3-intrastrand d(GpTpG)-Cisplatin cross-link

Purified 24-mer oligonucleotide containing a unique GTG sequence (5'-TCT TCT TCT **GTG **CAC TCT TCT TCT-3') was allowed to react at a concentration of 1 mM with a 3-fold molar excess of Cisplatin (3 mM) for 16 h at 37°C in a buffer containing 3 mM NaCl, 0.5 mM Na_2_HPO_4 _and 0.5 mM NaH_2_PO_4 _[48]. The purification of the platinated oligo was done by using 20% preparative denaturing polyacrylamide gel. The oligonucleotides were visualized using a hand-held UV lamp (254 nm) after placing the appropriate region of the gel onto TLC plate. The desired platinated oligonucleotide was excised, crushed and suspended in 1 ml H_2_O. The suspension was incubated overnight with agitation. The supernatant containing the platinated oligo was lyophilized and purified using Sephadex G25 column.

### Synthesis and purification of covalently closed circular DNA (cccDNA)

Covalently closed circular DNA containing a single 1,3-intrastrand d(GpTpG)-Cisplatin cross link (pt-GTG) was produced by priming 30 μg of plus strand M13 mp18 DNA modified to contain a sequence complementary to the platinated oligonucleotide within the polycloning site [48] with a 5-molar excess of 5'-phosphorylated platinated oligonucleotide in a 200-μl reaction mixture containing 10 mM Tris-HCl (pH7.9), 50 mM NaCl, 10 mM MgCl_2_, 1 mM DTT, 600 μM each of dATP, dCTP, dGTP and TTP, 2 mM ATP, 60 units of T4 DNA polymerase and T4 ligase (New England Biolab) for 4 h at 37°C. Closed circular DNA was isolated by CsCl/EtBr density gradient centrifugation and purified by consecutive butanol extraction, centrifugation in cetricon-10 microconcentrator (Amicon) and a Sephadex G-25 column (Sigma). DNA substrates were stored at 80°C in 10 mM Tris-HCl, 1 mM EDTA pH 8.0.

### Dual incision assay

Ten μl reaction mixture contain 19 μg cell extract, 32 ng pt-DNA, 5 mM MgCl_2_, 40 mM HEPES-KOH pH 7.8, 0.5 mM Dithiothreitol, 2 mM ATP, 23 mM phosphcreatine, 18 μg bovine serum albumin (BRL, nuclease free). The reaction mixtures were incubated for a further 30 min. To analyze the release of DNA containing the lesion, a 34-mer oligonucleotide is used [49] as a template by sequanase to incorporate radiolabeled dCTP on the 3' end of the excised fragment then the excised labelled fragments were analyzed on 14% polyacrylamide gel.

## Results

### HBx expression modulates the UV survival profile of Chang liver cells

**Figure 1:**
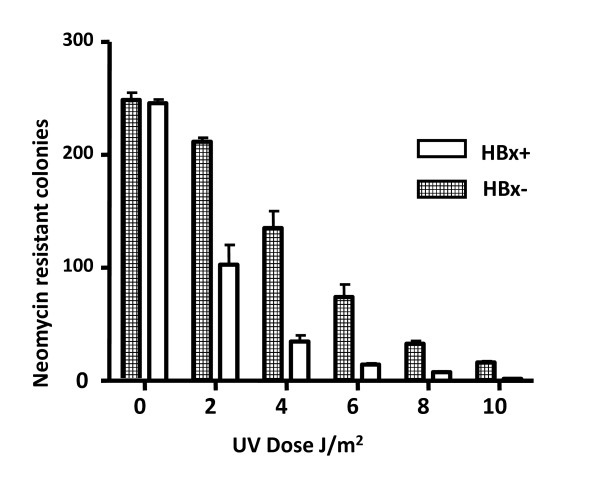
**UV survival profile of HBx expressing human liver cells**. HBx expression plasmid pSBDR and UV-damaged pRC/CMV were co transfected into chang liver cells. Plates were incubated in dark for 2 weeks in the presence of G418. The number of G418 resistant cells per 10^5 ^cells is plotted. Live cells were counted by staining with trypan blue prior to transfection. The ordinate represents the survival fraction, while the abscissa displays the dosage of UV irradiation. Each bar represents Mean ± S.D. from three independent experiments.

The effect of HBx expression on repair efficiency of a UV-damaged DNA in the human liver cell was monitored. HBx expressing plasmid pSBDR and a neomycin resistant plasmid pRC/CMV (control) were co-transfected into Chang liver cells. In the plasmid pSBDR, the HBx coding sequences are placed under the transcriptional control of native promoter and enhancer. pRC/CMV DNA was UV damaged for 2, 6, and 8 and 10 J/m^2 ^of UV radiation. As a control, UV-damaged pRC/CMV DNA was co-transfected along with a plasmid pHEN100 lacking the coding sequences of HBx. Cells were counted prior to co-transfection and selected in media containing G-418 for 2 weeks. Thereafter, G-418 resistant clones were counted. A decrease in the number of G-418 resistant clones per 10^5 ^cells was observed in HBx expressing cells when compared with non-expressing cells (Figure 1).

### HBx mutants fail to interact with TFIIH

**Figure 2:**
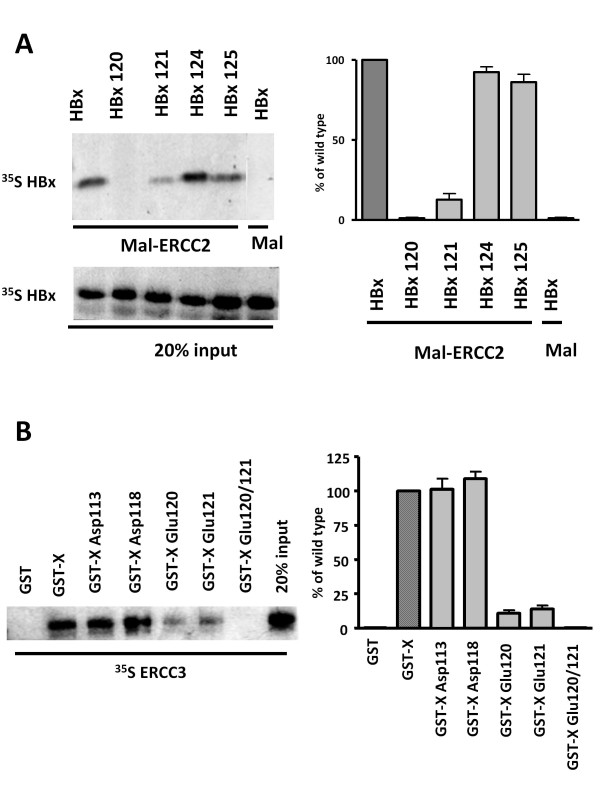
**HBx 120 and 121 mutants fail to interact with ERCC2 and ERCC3 components of human TFIIH**. **(A) **HBx and HBx mutants 120, 121, 124, and 125 were in vitro translated in the presence of ^35^S methionine and allowed to interact with the fusion protein of Mal-ERRCC2. Bound fractions are shown. **(B) **ERCC3 was in vitro translated in the presence of ^35^S-[methionine] and allowed to interact with GST (lane 1), GST-X (lanes 2), or GST HBx mutants Asp 113 (lane 3), Asp 118 (lane 4), Glu 120 (lane 5), Glu121 (lane 6) and double mutant Glu 120/121 (lane7).

We previously reported interactions between HBx and two components of TFIIH, ERRC2 and ERCC3 [28]. We identified a domain spanning aa 110-143, sufficient for these interactions between HBx and ERCC2 and ERCC3 [25] is domain was shown to be sufficient to stimulate the DNA helicase activity of purified TFIIH [25]. To identify the critical amino acids required for TFIIH interactions and associated functions, the conserved negatively charged residues in this domain were selected for mutagenesis studies. Using site-directed mutagenesis technique, individual amino acid residues, Asp 113, Asp 118, Glu 120, Glu 121, Glu 124 and Glu 125 were changed to non-polar Val. These HBx mutants were employed for interaction between HBx and ERCC2 and ERCC3. ERCC2 protein was expressed in *E. Coli *as a Maltose-ERCC2 fusion protein. Bacterial cellular extracts were immobilized on amylose resin. In this experiment the wild type HBx was in vitro translated and allowed to interact with either Mal-ERCC2 resin or with amylose beads alone. While HBx interacted with ERCC2 (Figure 2A, lane 1), no interaction was seen with amylose resin alone (Figure 2A, lane 6). In vitro translated ^35^S[methionine]-labeled HBx mutants Glu 120, Glu 121, Glu 124, and Glu 125 proteins were allowed to interact with Mal-ERCC2 (Figure 2A, lanes 2-5). The results of this analysis show that HBx mutant Glu 120 and Glu 121 did not interact with Mal-ERCC2 at any significant level (lanes 2 and 3). HBx mutants Glu 124 (lane 4) and Glu 125 (lane 5) showed only a modest reduction in binding to ERCC2 (see densitometric analysis in the right panel of Figure 2A).

To map the critical residue required for the interaction of HBx with ERCC3, GST pull down assay was performed in which ERCC3 proteins were synthesized in vitro in the presence of ^35^S[methionine] and allowed to interact with GST-fusion protein of HBx (Figure 2B). While wild type HBx interacted with ERCC3 (lane 2), no interactions were seen with GST (lane 1). HBx's mutants Asp 113 (lane 3) and Asp 118 (lane 4) showed normal interaction with ERCC3. On the other had HBx's mutant Glu 120, Glu 121 showed a reduction in binding to ERCC3 (lane 5 and 6). No interaction has been seen with the double mutant Glu 120/121 (lane 7). Collectively, these studies are consistent in identifying the Glu 120 and Glu 121 of HBx as critical residues involved in interactions with both DNA helicase components of TFIIH. These HBx mutant constructs provide a stronger evidence for the specificity of our previous resorts for the protein-protein interactions.

### HBx mutants fail to interact with TFIIH

**Figure 3:**
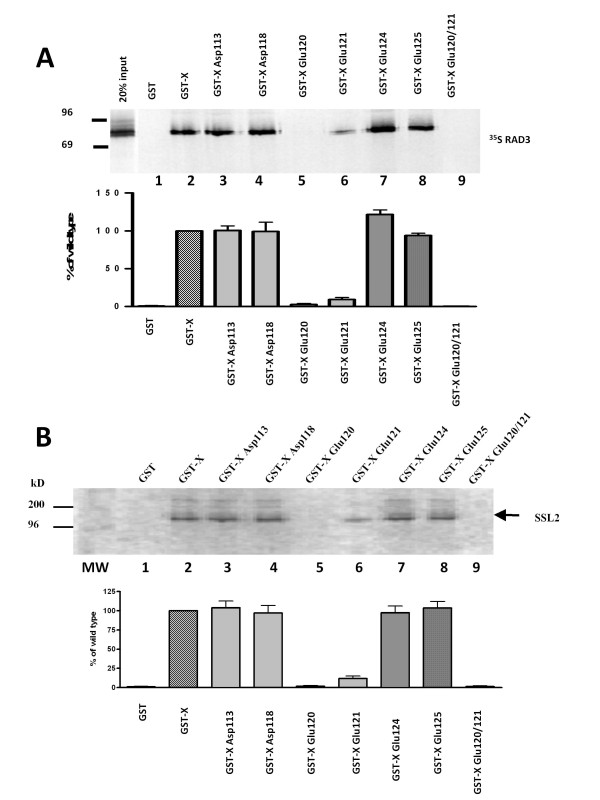
**Reduced interaction of HBX mutants with RAD3 (ERCC2 homolog) and SSL2 (ERCC3 homolog) components of yeast TFIIH**. **(A) **RAD3 was in vitro translated in the presence of ^35^S methionine and allowed to interact with GST (lane 1) or GST-X (lane 2), GST-XAsp113 (lane 3), GST-X Asp 118, (lane 4) GST-XGlu120 (lane 5), GST-X Glu121 (lane 6), GST-X Glu 124 (lane 7), GST-XGlu 125 (lane 8) and GST-X Glu 120/21 (lane 9). **(B) **SSL2 was synthesized in vitro and labeled with ^35^S methionine and allowed to interact with GST (lane 1) or GST-X (lane 2), GST-XAsp113 (lane 3), GST-X Asp 118, (lane 4) GST-XGlu120 (lane 5), GST-X Glu121 (lane 6), GST-X Glu 124 (lane 7), GST-XGlu 125 (lane 8), and GST-X Glu 120/21 (lane 9).

The HBx mutants were tested for their ability to physically interact with the DNA helicase components of yeast TFIIH (yTFIIH). The RAD3 and SSL2 represent the homologues of ERCC2 and ERCC3 components of mammalian TFIIH. In the first experiment, ^35^S-[methionine]-labelled wild type RAD3 component of yTFIIH was allowed to interact with glutathione affinity beads immobilized with either glutathione S-transferase (GST) or GST-HBx^wt ^or GST-HBx^mut ^fusion proteins which were extracted from bacteria (Figure 3A). After extensive washing, the bound proteins were analyzed by SDS-PAGE. In this analysis only HBx mutant Glu 120 failed to interact with RAD3 (Figure 3A, lane 6). Other mutants either interacted modestly or functioned as wild type HBx (Figure 3A).

Next, we also employed ^35^S[methionine]-labelled SSL2 homology of ERCC3 for its ability to interact with GST-X mutant proteins immobilized on GST affinity beads (Figure 3B). Consistent with Figure 3A, the results of these interaction studies identified Glu 120 as a critical residue for interaction with both components of yTFIIH.

### HBx expressing yeast cells modulates the UV survival profile

**Figure 4:**
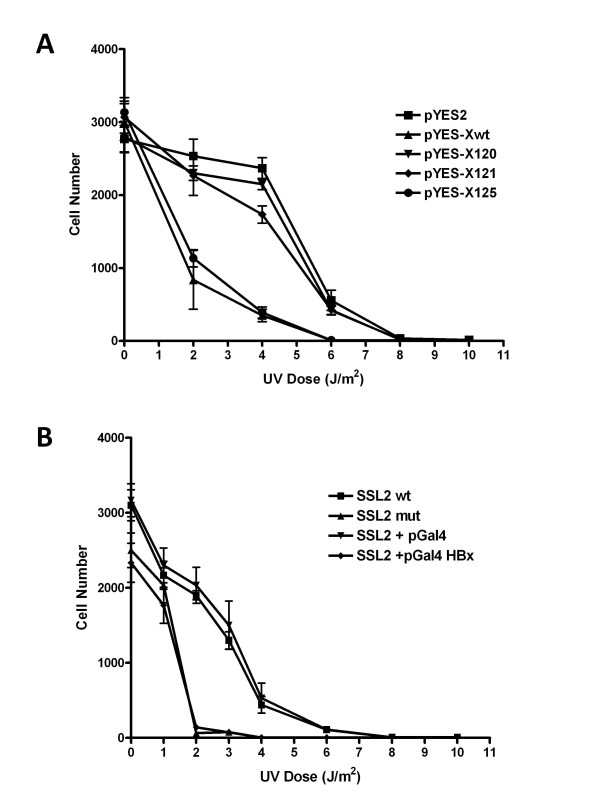
**HBx expression increases the UV sensitivity of yeast cells**. **(A) **UV survival profile of HBx expressing yeast cells. Saturated yeast cultures of strain 334 containing plasmids, pYES and pYES-X^wt ^and pYES-X^muts ^(as indicated), were diluted in water and plated on YMIN plates containing 2% glucose, 2% glycerol, 2% ethanol and 2% galactose (for induction of HBx). Cells were immediately irradiated under a germicidal lamp. Plates were then incubated in dark for at least 24 hrs and shifted to 30°C. Colonies were counted to determine the survival fraction. This is the average of three experiments. The ordinate represents the survival fraction, while the abscissa displays the dosage of UV irradiation. **(B) **UV survival profile of HBx expression in TFIIH mutant yeast cells. This is the average of three experiments. The ordinate represents the survival fraction, while the abscissa displays the dosage of UV irradiation.

To further correlate the effect of HBx associations with TFIIH, we employed a UV hypersensitivity assay as described by Gulyas and Donahue [50]. These authors have generated a SSL2 mutant (Ssl2-xp) that mimics the ERCC3 defect found in XP patients. This non-lethal mutant allele of SSL2 was shown to increases the sensitivity of yeast to UV irradiation when tested in an in vivo assay for viability. Upon UV irradiation of yeast, in which Ssl2-xp was the sole copy, 10^3 ^more cells died when compared to wild type, suggesting a direct correlation between defects in DNA repair enzymes and UV hypersensitivity. Using this assay system, the influence of HBx on DNA repair process in yeast was examined. HBx^wt ^and selected HBx^mutants ^were cloned in the yeast plasmid pYES with a selectable marker (Ura3) in which X is under the control of inducible galactose promoter. Yeast strain that lacks the galactose repressor (GAL-80) was used to transform and subjected to UV irradiation assay as described [50]. The results of UV irradiation experiment shown in Figure 4A, clearly suggest that yeast expressing HBx displayed an increased UV hypersensitivity. Since, we earlier showed that HBx interacts with SSL2 and RAD3 component of TFIIH [25], it is conceivable that the interactions between HBx and SSL2 and/or RAD3 are reflected in the impediment of cellular DNA repair process. To address this issue, HBx point mutants were employed. HBx mutants Glu 120, 121, 124, and 125 were transformed into yeast and assayed for UV hypersensitivity assay. HBx^mut120 ^which fails to interact with human and yeast TFIIH failed to influence the DNA repair in yeast (Figure 4A). The expression of HBx^mut ^proteins in yeast cells was confirmed by Immunoblotting. In all cases, similar levels of HBx expression were observed (data not shown). The results of the UV hypersensitivity assay are consistent with the hypothesis that the inability of the HBx to interact with TFIIH directly correlates with its inability to impede the DNA repair process.

We next asked the question, does the expression of HBx in the mutant yeast strain lacking the carboxyl-terminus of SSL2 (ERCC3 homologue) affect the UV survival profile? A mutant yeast strain with a deletion of 79aa in the carboxyl terminus of was used in the UV-hypersensitivity experiment [50]. The deletion in ssl2 strain overlaps with the ERCC3 deletion mutant that contains the ATPase activity and does not interact with HBx (data not shown). The yeast strain was transformed with plasmid pGal4-X^wt^. In the UV hypersensitivity experiment, HBx did not affect the survival profile of the mutant yeast strain with C-terminal deletion of SSL2 (Figure 4b). These results suggest that TFIIH regulated pathway is utilized by HBx in the impediment of the DNA repair process and that HBx-TFIIH physical interaction is crucial to influence this process.

### Effect of HBx expression on in vitro DNA repair reaction

Next, we assayed the efficiency of HBx expressing yeast cell lysates to repair UV-damaged plasmid DNA. This was compared with non-expressing and HBx mutant expressing cell lysates. Wang and co-workers [47] developed a fairly simple and effective assay to monitor DNA repair in vitro. This assay relies on the repair synthesis of a plasmid which has been previously treated with a base-damaging agent N-acetoxy-2-acetylaminofluorene (AAAF) or UV irradiation. Damaged plasmids are incubated with wild type yeast cell-free extracts and ^32^P-labeled dCTP. Radioactivity incorporated into the damaged plasmid during DNA repair is observed by agarose gel electrophoresis followed by autoradiography. By employing the mutant alleles of RAD3 and SSL2, Wang and co-workers [47] were able to define a functional role for yeast TFIIH in DNA repair. We employed this assay to determine the effect of HBx on DNA repair process in vitro. To control the specificity of in vitro DNA repair reaction, we also used TFIIH (ssl2) mutant and NER defective rad 1 and rad51 deletion yeast strains as controls.

First, UV irradiated plasmid pBR322 was subjected to DNA repair in vitro, with extracts of wild type yeast strain 334 and those transformed with pYES-2 (vector alone), pYES-X (HBx expressing vector) and its mutants Glu 120, Glu 121, Glu 124 and Glu 125. Un-irradiated plasmid pUC18 DNA was used as a control. Yeast lysates were prepared 16 hr after treatment with 2% galactose for the expression of HBx and its mutant proteins. HBx and its mutant proteins were expressed equally in these yeast strains as confirmed by Western blotting (data not shown).

**Figure 5:**
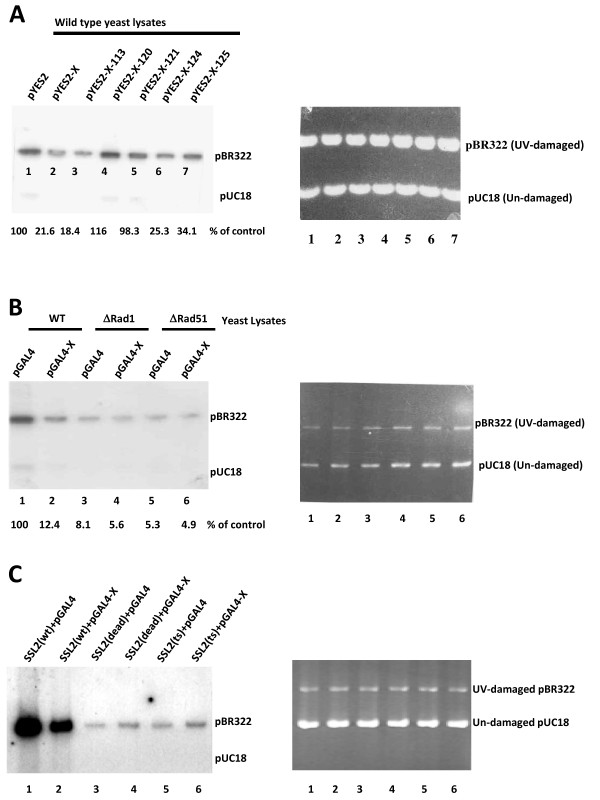
**HBx impedes the DNA repair of UV damaged plasmid DNA in-vitro**. **(A) **In vitro repair of UV-damaged pBR322 DNA using yeast lysates expressing HBx and its mutants. The repair reaction contained, 0.3 μg un-irradiated pUC18 and 0.3 μg UV-irradiated pBR322 substrate, was performed as discussed in the experimental procedure. Control plasmid (lane 1); HBx expressing plasmid (lane 2); and its mutant Glu120 (lane 3); Glu 121 (lane 4); Glu 124 (lane 5) and Glu 125 (lane6). Reactions were incubated for 6 hours at 30°C. Reactions were stopped by the addition of EDTA and then incubated with RNAse, SDS and proteinase K. Plasmids were digested with HindIII and loaded on 1% agarose gel. After overnight electrophoresis, the gel was photographed under near-UV transillumination with Polaroid film (right panel) and an autoradiograph of the dried gel was obtained (left panel) **(B) **HBx is unable to repair the damaged plasmid DNA in Rad1 and Rad51 mutant yeast strain. Plasmid p-GAL4 and pGAL4-X were transformed into yeast strains with normal RAD1 and RAD51 genes (lane 1, 2), with deletion of Rad1 (lane 3, 4) and with deletion of RAd51 (lane 5-6). Nuclear extract were assayed for DNA repair of UV-damaged pUC18 DNA **(C) **HBx is unable to repair damaged plasmid DNA in SSL2 mutant (dead) and temperature sensitive yeast strain. Plasmid p-Gal4 and pGAL4-X were transformed into yeast strains with normal SSL2 (lane 1, 2) mutant SSL2-dead strain (lane 3, 4) and temperature strain (lane 5-6). Nuclear extracts were assayed for DNA repair of UV-damaged pBR322 DNA The yeast **ts **strain was grown at room temperature (20-21°C).

Figure 5A shows the results of this experiment. The repair synthesis of UV irradiated plasmid pUC18 using the yeast crude extracts transformed with vector alone (lane 1), HBx expressing vector, (lane 2) and HBx mutants Glu 120 (lane 3), Glu 121 (lane 4), Glu 124 (lane 5) and Glu 125 (lane 6). The incorporation of ^32^P[dCTP] as a measure of DNA repair is shown in Figure 5. These results clearly suggest that HBx expressing yeast lysates are defective in repairing the UV-damaged DNA in vitro (compare lane 1 with lane 2). HBx mutant Asp 113 that has retained the ability to interact with TFIIH (Figure 2A-C) also retains the ability to impede the DNA repair process like wild type HBx (lane 3). Yeast lysates expressing other mutants of HBx showed varying degrees of DNA repair efficiencies (lanes 4-7). More importantly, HBx's mutant Glu 120 which failed to interact with TFIIH also failed to influence the repair process in vitro (lane 3). The results shown in Figure 5A are encouraging, as no incorporation in the un-damaged pBR322 DNA was observed. To further confirm that non-specific incorporation of radioactivity has not occurred in this reaction, we used HBx expressing NER defective yeast lysates. Two mutant yeast strains with deletions in Rad-1 and Rad-51 were transformed with HBx expressing plasmid pGAL4-X and a control plasmid pGAL4. Yeast lysates were prepared after 16 hrs induction with 2% glacotose and subjected in repair reaction (Figure 5B, lanes 3-6). Both Rad-1 and Rad-51 NER defective lysates showed no incorporation (lanes 3 and 5). HBx expression in these mutant yeast lysates had no effect on the repair reaction (lane 4 and 6). This suggests that indeed specific DNA repair reaction has occurred in Figure 5A. These results are consistent with the hypothesis that HBx expressing wild type yeast lysates have diminished DNA repair efficiency of UV-damaged plasmid DNA.

Next, we examined the ability of HBx to alter DNA excision repair reaction in a TFIIH mutant yeast strain (Figure 5C). Wild type yeast strain and two TFIIH mutant yeast strains ssl2 (dead) and ssl2 (ts) [37] were transformed with a control plasmid pGAL4 and HBx expressing pGAL4-X DNAs. Yeast lysates were prepared as described. UV-damaged pBR322 DNA was used. Consistent with our previous results, HBx expression in wild type strain diminished the ability to repair the DNA (lane 2). TFIIH mutant yeast lysates with HBx (lane 4 and 6) or without HBx (lanes 3 and 5) were equally deficient in DNA repair synthesis, suggesting that HBx impinge its influence on DNA repair via TFIIH. In summary, using myriad experimental strategies, our results implicate HBx in DNA repair process via its physical interactions with the helicase components of TFIIH.

### HBx protein inhibits excision of damaged DNA in the dual incision assay

**Figure 6:**
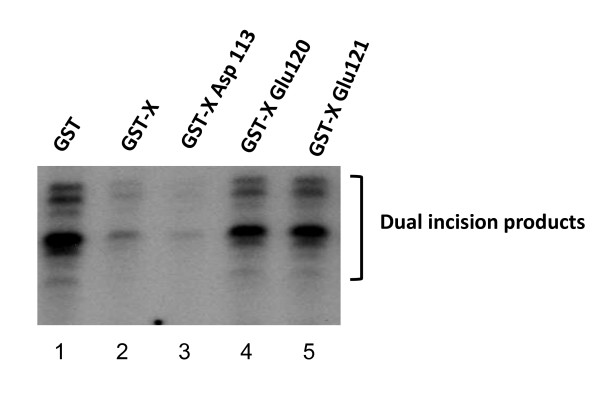
**HBx protein inhibits excision of damaged DNA in dual incision assay**. Measurement of the effect of X protein on the dual excision of the Damaged DNA using 40 μg of HeLa whole cell extract and 20 ng of Pt-DNA. GST (lane 1) or GST-X (lane 2), GST-XAsp113 (lane 3), GST-XGlu120 (lane 4), GST-X Glu121 (lane 5).

To measure the effect of X protein on the excision of the Damaged DNA, we used 40 μg of HeLa whole cell extract and 20 ng of Pt-DNA. Figure 6 shows that HBx or HBx 113 mutant but not HBx120 or HBx121 is able to inhibit the excision of the platinated fragment.

## Discussion

HBx protein has been proposed to play a role in the development of HCC. HBx has been shown to possess pleiotropic functions including impairment of cell cycle progression [51], interaction with transcription machinery [9-13], and cell signal transduction and apoptosis mechanisms [29,52-54]. Furthermore, HBx associated physically with p53 resulting in the sequestration of p53 in the cytoplasm (28), inhibition of p53 function including its DNA binding and transactivation activities [55] as well as p53 interaction with XPB protein [55]. Several studies suggested a potential role of HBx cellular DNA repair process. This is borne out by its associations with TFIIH [25,28], a probable DNA repair factor UV-DDB [23,42,56], p53 tumor suppressor protein [55,57], ss-DNA [36], and UV-damaged DNA [58,59].

### HBx expression inhibit DNA repair

Our study provides evidence that HBx can inhibit DNA repair pathway. In the absence of UV damage, cells expressing HBx were found to be similar to control cells in cell growth measured by colony formation assay (Figure 1). Similar observations were reported by Lee and co-workers [60]. They demonstrated that HBx expression did not affect the morphology, viability, and cell cycle/apoptosis profiles or DNA repair machinery of UV-untreated HepG2 cells. However, HBx-expressing cells exhibited increased sensitivity to UV damage and reduced DNA repair capacity. It has been shown that mice carrying HBx as a transgene show a direct correlation between the level of HBx expression and the likelihood to develop HCC [61,62]. However certain lineages of HBx transgenic mice do not exhibit tumour development unless coupled with other factors such as exposure to the hepatocarcinogen diethylnitrosamine [63] or when combined with *c-myc *induction [64]. It has been suggested previously that HBx does not directly cause cancer but plays a role in liver oncogenesis as a cofactor or tumour promoter [60]. Chronic HBV infection may present a long-term opportunity for an initiating event to occur, and HBx may act by modifying cellular regulatory/control mechanisms facilitating the culmination of the transformation process in the cell. In this regard, a highly probable tumour-initiating event is DNA damage.

### HBx mutants failed to interact with TFIIH

We continue to characterize the specific domains of HBx involved in affecting the DNA repair process. We have employed approaches to elucidate the mechanism(s) by which HBx interactions with DNA excision repairs factor TFIIH could lead to impaired DNA repair. DNA repair system is the primary defence against accumulation of mutations in genomic DNA and activation of cellular carcinogenesis. Deficiencies in DNA repair pathways have been linked to common cancer predisposition syndromes. Notable among these are the hereditary nonpolyposis colorectal cancer (HNPCC) and skin cancer or xeroderma pigmentosum [46,65]. DNA repair occurs by kinetically two different pathways: one involved with repair of the overall genome (global repair) and one involved with repair of transcribed genes (transcription coupled-repair) [46,66,67]. Studies have demonstrated that some of the essential DNA repair proteins in yeast and mammalian cells are a part of basal transcription factor TFIIH [26,67,68]. In humans, the defects in XPD/ERCC2 and XPB/ERCC3 genes lead to xeroderma pigmentosum (XP) [69] and Cockayne's Syndrome (CS) [65,66]. Both conditions are manifested by the inability of the cells to efficiently repair damaged DNA. In yeast, RAD3 and SSL2 (RAD25) are the homologues of XPD/ERCC2 and XPB/ERCC3 respectively. These genes are essential both in yeast and mammals.

Since TFIIH is one of the minimal set of factors required for transcription initiation and DNA excision repair, the association of HBx implicates a fundamental role in the processes affected by HBx [70,71]. A large body of data, supports the transcriptional transactivation role of HBx [11,72,73]. It remains to be determined if HBx's ability to stimulate DNA helicase activity of ERCC2/ERCC3 [25] is functionally relevant to both DNA repair and transcription initiation.

### Mapping of the functional domain of HBx

Many studies showed that HBx plays an important role in HCC pathogenesis by interacting with cellular oncogenes [21-23] and that its functional domain involved in oncogenesis is at the middle of HBx protein [24,25]. Several studies have also shown that HBx can induce apoptosis [26-29].

Tang and co-worker has mapped the coactivation domain within the C-terminal, two thirds of which (aa51-138) is identified to that of the transactivation. In contrast, the N-terminal of HBx has the ability to down regulate transactivation and was defined as the negative regulatory domain [74].

It has been shown recently that the COOH-terminal truncated HBx plays a critical role in the HCC carcinogenesis via the activation of cell proliferation [75]. Alteration of HBV X gene has been detected more frequently in tissue samples of cirrhosis and/or HCC than in those of mild liver disease [76]. However, the mechanism of HBx in HCC carcinogenesis is still unclear, although many studies have associated it to ability of HBx *trans*-activating cellular oncogenes and signaling cascades that stimulate cell proliferation and lead to HCC carcinogenesis [1,17,77-79]. It has been demonstrated that the full-length HBx contains two function domains: oncogenic domain (the NH2 terminal through middle peptide) and proapoptotic domain (the COOH-terminal peptide). There is a balance between these two functions in HBV-infected hepatocytes. When the proapoptotic domain is deleted by an unknown mechanism during the viral integration, the balance is broken and the oncogenic function becomes dominant, leading to the subsequent development of HCC.

HBx has been shown to enhance cell susceptibility to cytotoxic effect of genotoxic agents, e.g. UVC and aflatoxins, that induce bulky adducts. This effect has been linked to impaired regulation of DNA repair and associated cell cycle checkpoint mechanisms [24-27], and/or the proapoptotic effect of HBx [45]. DNA damage induced by bulky adducts are preferred substrates for NER mechanism, where the TFIIH repair complex plays an essential role [30]. Inhibition of TFIIH activity by HBx may inhibit DNA repair and hence promote cells to undergo apoptosis. While several studies have focused on the transactivation capacity of the HBx protein in carcinogenesis, our data indicates that HBX is capable of transcriptional repression while maintaining it transactivation functions on NF-kB and AP1 responsive elements. The implication of transactivation in carcinogenesis is demonstrated primarily in transient systems and there is evidence that HBx-induced transactivation is not sufficient for cell transformation [47]. The observation that HBx suppresses XPB and XPD in liver tissue from HBx-transgenic mice supports the biological relevance of our findings. XPB and XPD helicase and ATPase activities, but not the TFIIH kinase, are required for NER function [30-33].

Previous studies have shown that HBx inactivate the p53 tumour suppressor protein and impair DNA repair, cell cycle, and apoptosis mechanisms. HBx was shown to represses two components of the transcription-repair factor TFIIH, XPB (p89), and XPD (p80), both in p53-proficient and p53-deficient liver cells. This inhibition is observed while HBx maintains its transactivation function. Expression of HBx in liver cells results in down-regulation of endogenous XPB and XPD mRNAs and proteins. In liver tissue from HBx transgenic, XPB and XPD proteins are down-regulated in comparison to matched normal liver tissue [48]. HBx expression on hepatocytes nucleotide excision repair has been successfully studied in primary wild-type and *p53*-null mouse hepatocytes. Transient HBx expression reduces global DNA repair in wild-type cells to the level of *p53*-null hepatocytes and has no effect on the repair of a transfected damaged plasmid [53]. Inhibition of p53-mediated apoptosis by HBx may provide a clonal selective advantage for hepatocytes expressing this integrated viral gene during the early stages of human liver carcinogenesis [54].

To date, a few mechanisms of HBV-induced HCC have been proposed. Early studies proposed that insertional mutagenesis of the HBV genome into human chromosomes might cause inactivation of tumor suppressor/proto-oncogenes [80-82]. However, later studies have shown that integration of HBV genome is genome-wide and unlikely attacks a specific tumor suppressor or proto-oncogene [82,83]. HBx initiates transactivation as well as induction of signal transduction pathways such as Ras/Raf-1 [84,85]. The large surface protein has been shown to induce HCC in the transgenic mouse model [86,87]. Our results are consistent to the hypothesis that HBx impedes the DNA repair via interaction with TFIIH. In the dual incision assay HBx120 or HBx121 mutants fail to impede the repair process. These two residues seem to be critical determinant in DNA repair in HBx mediated inhibition as two mutants fail to interact with TFIIH.

## Conclusions

In our study, we defined an inhibitory role of HBx in DNA excision repair process, thus hampering the cellular ability to repair the damaged DNA more effectively during HBx expression. Recent studies on HCC in Taiwan, the pre-S_1_/S_2 _mutant were shown to induce oxidative stress and DNA damage in Ground glass hepatocytes (GGHs), the pathological hallmarks for late phases of chronic HBV infection [88]. Other studies have reported that a defect in the *ogg1 *DNA repair gene is involved in various types of human carcinogenesis [89]. Therefore, efficient DNA repair for damaged DNA should play an important role in cancer prevention. Our findings suggest that HBx may act as the promoting factor by inhibiting DNA repair causing DNA damage and accumulation of errors, thereby contributing to HCC development.

## List of abbreviations

The abbreviations used are, (HBV): hepatitis B virus; (TFIIH): transcription factor IIH; (ERCC): excision repair cross complementing; (NER): nucleotide excision repair; (XP): xeroderma pigmentosum.

## Competing interests

The authors declare that they have no competing interests.

## Authors' contributions

IQ conceived the idea coupled with the design and execution of experiments and have also written the manuscript. KF and HAH performed Dual incision assay, in-vitro experiments, prepared Figures and edited the manuscript. The financial support was provided by grants to IQ and HAH.

## References

[B1] Neuveut C Wei Y Buendia MA Mechanisms of HBV-related hepatocarcinogenesis J Hepatol 2010 52 4 594 604 10.1016/j.jhep.2009.10.033 20185200

[B2] Fung J Lai CL Yuen MF Hepatitis B and C virus-related carcinogenesis Clin Microbiol Infect 2009 15 11 964 970 10.1111/j.1469-0691.2009.03035.x 19874379

[B3] Benhenda S Cougot D Buendia MA Neuveut C Hepatitis B virus X protein molecular functions and its role in virus life cycle and pathogenesis Adv Cancer Res 2009 103 75 109 full_text 19854353 10.1016/S0065-230X(09)03004-8

[B4] Bruni R Conti I Villano U Giuseppetti R Palmieri G Rapicetta M Lack of WHV integration nearby N-myc2 and in the downstream b3n and win loci in a considerable fraction of liver tumors with activated N-myc2 from naturally infected wild woodchucks Virology 2006 345 1 258 269 10.1016/j.virol.2005.09.061 16271377

[B5] Park EH Koh SS Srisuttee R Cho IR Min HJ Jhun BH Lee YS Jang KL Kim CH Johnston RN Expression of HBX, an oncoprotein of hepatitis B virus, blocks reoviral oncolysis of hepatocellular carcinoma cells Cancer Gene Ther 2009 16 5 453 461 10.1038/cgt.2008.95 19096445

[B6] Mukherji A Janbandhu VC Kumar V HBx protein modulates PI3K/Akt pathway to overcome genotoxic stress-induced destabilization of cyclin D1 and arrest of cell cycle Indian J Biochem Biophys 2009 46 1 37 44 19374252

[B7] He Y Sun HQ He XE Wang WL Lei JH Knockdown of HBx by RNAi inhibits proliferation and enhances chemotherapy-induced apoptosis in hepatocellular carcinoma cells Med Oncol 2009 10.1007/s12032-009-9363-0 19949899

[B8] Cheng P Li Y Yang L Wen Y Shi W Mao Y Chen P Lv H Tang Q Wei Y Hepatitis B virus X protein (HBx) induces G2/M arrest and apoptosis through sustained activation of cyclin B1-CDK1 kinase Oncol Rep 2009 22 5 1101 1107 19787227 10.3892/or_00000542

[B9] Cheng B Guo X Zheng Y Wang Y Liu C Li P The effects of HBx gene on the expression of DNA repair enzymes hOGG1 and hMYHalpha mRNA in HepG2 cells J Huazhong Univ Sci Technolog Med Sci 2009 29 2 187 192 10.1007/s11596-009-0210-5 19399402

[B10] Kuo CY Wang JC Wu CC Hsu SL Hwang GY Effects of hepatitis B virus X protein (HBx) on cell-growth inhibition in a CCL13-HBx stable cell line Intervirology 2008 51 1 26 32 10.1159/000118793 18309246

[B11] Butel JS Lee TH Slagle BL Is the DNA repair system involved in hepatitis-B-virus-mediated hepatocellular carcinogenesis? Trends Microbiol 1996 4 3 119 124 10.1016/0966-842X(96)81529-0 8868091

[B12] Nassal M Schaller H Hepatitis B virus replication Trends Microbiol 1993 1 6 221 228 10.1016/0966-842X(93)90136-F 8137119

[B13] Han M Yan W Guo W Xi D Zhou Y Li W Gao S Liu M Levy G Luo X Hepatitis B virus-induced hFGL2 transcription is dependent on c-Ets-2 and MAPK signal pathway J Biol Chem 2008 283 47 32715 32729 10.1074/jbc.M806769200 18801734

[B14] Kang-Park S Lee JH Shin JH Lee YI Activation of the IGF-II gene by HBV-X protein requires PKC and p44/p42 map kinase signalings Biochem Biophys Res Commun 2001 283 2 303 307 10.1006/bbrc.2001.4767 11327698

[B15] Choi CY Choi BH Park GT Rho HM Activating transcription factor 2 (ATF2) down-regulates hepatitis B virus X promoter activity by the competition for the activating protein 1 binding site and the formation of the ATF2-Jun heterodimer J Biol Chem 1997 272 27 16934 16939 10.1074/jbc.272.27.16934 9202004

[B16] Li B Gao B Ye L Han X Wang W Kong L Fang X Zeng Y Zheng H Li S Hepatitis B virus X protein (HBx) activates ATF6 and IRE1-XBP1 pathways of unfolded protein response Virus Res 2007 124 1-2 44 49 10.1016/j.virusres.2006.09.011 17092596

[B17] Maguire HF Hoeffler JP Siddiqui A HBV X protein alters the DNA binding specificity of CREB and ATF-2 by protein-protein interactions Science 1991 252 5007 842 844 10.1126/science.1827531 1827531

[B18] Cheong JH Yi M Lin Y Murakami S Human RPB5, a subunit shared by eukaryotic nuclear RNA polymerases, binds human hepatitis B virus X protein and may play a role in X transactivation EMBO J 1995 14 1 143 150 7828586 10.1002/j.1460-2075.1995.tb06984.x PMC398061

[B19] Le TT Zhang S Hayashi N Yasukawa M Delgermaa L Murakami S Mutational analysis of human RNA polymerase II subunit 5 (RPB5): the residues critical for interactions with TFIIF subunit RAP30 and hepatitis B virus X protein J Biochem 2005 138 3 215 224 10.1093/jb/mvi119 16169872

[B20] Wang JH Yun C Kim S Chae S Lee YI Kim WH Lee JH Kim W Cho H Reactivation of p53 in cells expressing hepatitis B virus X-protein involves p53 phosphorylation and a reduction of Hdm2 Cancer Sci 2008 99 5 888 893 10.1111/j.1349-7006.2008.00754.x 18294283 PMC11159080

[B21] Park SG Min JY Chung C Hsieh A Jung G Tumor suppressor protein p53 induces degradation of the oncogenic protein HBx Cancer Lett 2009 282 2 229 237 10.1016/j.canlet.2009.03.019 19375220

[B22] Qadri I Maguire HF Siddiqui A Hepatitis B virus transactivator protein X interacts with the TATA-binding protein Proc Natl Acad Sci USA 1995 92 4 1003 1007 10.1073/pnas.92.4.1003 7862623 PMC42625

[B23] Bontron S Lin-Marq N Strubin M Hepatitis B virus X protein associated with UV-DDB1 induces cell death in the nucleus and is functionally antagonized by UV-DDB2 J Biol Chem 2002 277 41 38847 38854 10.1074/jbc.M205722200 12151405

[B24] Leupin O Bontron S Strubin M Hepatitis B virus X protein and simian virus 5 V protein exhibit similar UV-DDB1 binding properties to mediate distinct activities J Virol 2003 77 11 6274 6283 10.1128/JVI.77.11.6274-6283.2003 12743284 PMC154990

[B25] Qadri I Conaway JW Conaway RC Schaack J Siddiqui A Hepatitis B virus transactivator protein, HBx, associates with the components of TFIIH and stimulates the DNA helicase activity of TFIIH Proc Natl Acad Sci USA 1996 93 20 10578 10583 10.1073/pnas.93.20.10578 8855220 PMC38195

[B26] Jaitovich-Groisman I Benlimame N Slagle BL Perez MH Alpert L Song DJ Fotouhi-Ardakani N Galipeau J Alaoui-Jamali MA Transcriptional regulation of the TFIIH transcription repair components XPB and XPD by the hepatitis B virus x protein in liver cells and transgenic liver tissue J Biol Chem 2001 276 17 14124 14132 11278765 10.1074/jbc.M010852200

[B27] Seto E Mitchell PJ Yen TS Transactivation by the hepatitis B virus X protein depends on AP-2 and other transcription factors Nature 1990 344 6261 72 74 10.1038/344072a0 2154703

[B28] Haviv I Vaizel D Shaul Y pX, the HBV-encoded coactivator, interacts with components of the transcription machinery and stimulates transcription in a TAF-independent manner Embo J 1996 15 13 3413 3420 8670843 PMC451905

[B29] Chirillo P Pagano S Natoli G Puri PL Burgio VL Balsano C Levrero M The hepatitis B virus X gene induces p53-mediated programmed cell death Proc Natl Acad Sci USA 1997 94 15 8162 8167 10.1073/pnas.94.15.8162 9223332 PMC21574

[B30] Kim WH Hong F Jaruga B Zhang ZS Fan SJ Liang TJ Gao B Hepatitis B virus X protein sensitizes primary mouse hepatocytes to ethanol- and TNF-alpha-induced apoptosis by a caspase-3-dependent mechanism Cell Mol Immunol 2005 2 1 40 48 16212910

[B31] Yang Y Zheng L Lv G Jin X Sheng J Hepatocytes treated with HBV X protein as cytotoxic effectors kill primary hepatocytes by TNF-alpha-related apoptosis-induced ligand-mediated mechanism Intervirology 2007 50 5 323 327 10.1159/000106463 17657161

[B32] Yi YS Park SG Byeon SM Kwon YG Jung G Hepatitis B virus X protein induces TNF-alpha expression via down-regulation of selenoprotein P in human hepatoma cell line, HepG2 Biochim Biophys Acta 2003 1638 3 249 256 12878326 10.1016/s0925-4439(03)00090-5

[B33] Nakatake H Chisaka O Yamamoto S Matsubara K Koshy R Effect of X protein on transactivation of hepatitis B virus promoters and on viral replication Virology 1993 195 2 305 314 10.1006/viro.1993.1381 8337816

[B34] Chen HS Kaneko S Girones R Anderson RW Hornbuckle WE Tennant BC Cote PJ Gerin JL Purcell RH Miller RH The woodchuck hepatitis virus X gene is important for establishment of virus infection in woodchucks J Virol 1993 67 3 1218 1226 8437213 10.1128/jvi.67.3.1218-1226.1993 PMC237487

[B35] Zoulim F Saputelli J Seeger C Woodchuck hepatitis virus X protein is required for viral infection in vivo J Virol 1994 68 3 2026 2030 8107266 10.1128/jvi.68.3.2026-2030.1994 PMC236671

[B36] Qadri I Ferrari ME Siddiqui A The hepatitis B virus transactivator protein, HBx, interacts with single-stranded DNA (ssDNA). Biochemical characterizations of the HBx-ssDNA interactions J Biol Chem 1996 271 26 15443 15450 10.1074/jbc.271.26.15443 8663128

[B37] Giglia-Mari G Coin F Ranish JA Hoogstraten D Theil A Wijgers N Jaspers NG Raams A Argentini M van der Spek PJ A new, tenth subunit of TFIIH is responsible for the DNA repair syndrome trichothiodystrophy group A Nat Genet 2004 36 7 714 719 10.1038/ng1387 15220921

[B38] Hashimoto S Egly JM Trichothiodystrophy view from the molecular basis of DNA repair/transcription factor TFIIH Hum Mol Genet 2009 18 R2 R224 230 10.1093/hmg/ddp390 19808800

[B39] Scharer OD Hot topics in DNA repair: the molecular basis for different disease states caused by mutations in TFIIH and XPG DNA Repair (Amst) 2008 7 2 339 344 10.1016/j.dnarep.2007.10.007 18077223 PMC2246058

[B40] Seroz T Hwang JR Moncollin V Egly JM TFIIH: a link between transcription, DNA repair and cell cycle regulation Curr Opin Genet Dev 1995 5 2 217 221 10.1016/0959-437X(95)80011-5 7613092

[B41] Svejstrup JQ Wang Z Feaver WJ Wu X Bushnell DA Donahue TF Friedberg EC Kornberg RD Different forms of TFIIH for transcription and DNA repair: holo-TFIIH and a nucleotide excision repairosome Cell 1995 80 1 21 28 10.1016/0092-8674(95)90447-6 7813015

[B42] Lee TH Elledge SJ Butel JS Hepatitis B virus X protein interacts with a probable cellular DNA repair protein J Virol 1995 69 2 1107 1114 7815490 10.1128/jvi.69.2.1107-1114.1995 PMC188683

[B43] Aboussekhra A Biggerstaff M Shivji MK Vilpo JA Moncollin V Podust VN Protic M Hubscher U Egly JM Wood RD Mammalian DNA nucleotide excision repair reconstituted with purified protein components Cell 1995 80 6 859 868 10.1016/0092-8674(95)90289-9 7697716

[B44] Aboussekhra A Wood RD Detection of nucleotide excision repair incisions in human fibroblasts by immunostaining for PCNA Exp Cell Res 1995 221 2 326 332 10.1006/excr.1995.1382 7493631

[B45] Zhovmer A Oksenych V Coin F Two sides of the same coin: TFIIH complexes in transcription and DNA repair ScientificWorldJournal 2010 10 633 643 20419276 10.1100/tsw.2010.46 PMC5763819

[B46] Wood RD DNA repair in eukaryotes Annu Rev Biochem 1996 65 135 167 10.1146/annurev.bi.65.070196.001031 8811177

[B47] Wang Z Svejstrup JQ Feaver WJ Wu X Kornberg RD Friedberg EC Transcription factor b (TFIIH) is required during nucleotide-excision repair in yeast Nature 1994 368 6466 74 76 10.1038/368074a0 8107888

[B48] Moggs JG Szymkowski DE Yamada M Karran P Wood RD Differential human nucleotide excision repair of paired and mispaired cisplatin-DNA adducts Nucleic Acids Res 1997 25 3 480 491 10.1093/nar/25.3.480 9016585 PMC146461

[B49] Shivji MK Ferrari E Ball K Hubscher U Wood RD Resistance of human nucleotide excision repair synthesis in vitro to p21Cdn1 Oncogene 1998 17 22 2827 2838 10.1038/sj.onc.1202352 9879989

[B50] Gulyas KD Donahue TF SSL2, a suppressor of a stem-loop mutation in the HIS4 leader encodes the yeast homolog of human ERCC-3 Cell 1992 69 6 1031 1042 10.1016/0092-8674(92)90621-I 1318786

[B51] Benn J Schneider RJ Hepatitis B virus HBx protein deregulates cell cycle checkpoint controls Proc Natl Acad Sci USA 1995 92 24 11215 11219 10.1073/pnas.92.24.11215 7479968 PMC40602

[B52] Shintani Y Yotsuyanagi H Moriya K Fujie H Tsutsumi T Kanegae Y Kimura S Saito I Koike K Induction of apoptosis after switch-on of the hepatitis B virus X gene mediated by the Cre/loxP recombination system J Gen Virol 1999 80 Pt 12 3257 3265 10567659 10.1099/0022-1317-80-12-3257

[B53] Bergametti F Prigent S Luber B Benoit A Tiollais P Sarasin A Transy C The proapoptotic effect of hepatitis B virus HBx protein correlates with its transactivation activity in stably transfected cell lines Oncogene 1999 18 18 2860 2871 10.1038/sj.onc.1202643 10362257

[B54] Terradillos O Pollicino T Lecoeur H Tripodi M Gougeon ML Tiollais P Buendia MA p53-independent apoptotic effects of the hepatitis B virus HBx protein in vivo and in vitro Oncogene 1998 17 16 2115 2123 10.1038/sj.onc.1202432 9798683

[B55] Wang XW Forrester K Yeh H Feitelson MA Gu JR Harris CC Hepatitis B virus X protein inhibits p53 sequence-specific DNA binding, transcriptional activity, and association with transcription factor ERCC3 Proc Natl Acad Sci USA 1994 91 6 2230 2234 10.1073/pnas.91.6.2230 8134379 PMC43344

[B56] Hannan MA Hellani A Al-Khodairy FM Kunhi M Siddiqui Y Al-Yussef N Pangue-Cruz N Siewertsen M Al-Ahdal MN Aboussekhra A Deficiency in the repair of UV-induced DNA damage in human skin fibroblasts compromised for the ATM gene Carcinogenesis 2002 23 10 1617 1624 10.1093/carcin/23.10.1617 12376469

[B57] Al-Mohanna MA Al-Khodairy FM Krezolek Z Bertilsson PA Al-Houssein KA Aboussekhra A p53 is dispensable for UV-induced cell cycle arrest at late G(1) in mammalian cells Carcinogenesis 2001 22 4 573 578 10.1093/carcin/22.4.573 11285191

[B58] Capovilla A Carmona S Arbuthnot P Hepatitis B virus X-protein binds damaged DNA and sensitizes liver cells to ultraviolet irradiation Biochem Biophys Res Commun 1997 232 1 255 260 10.1006/bbrc.1997.6269 9125143

[B59] Al-Moghrabi NM Al-Sharif IS Aboussekhra A UV-induced de novo protein synthesis enhances nucleotide excision repair efficiency in a transcription-dependent manner in S. cerevisiae DNA Repair (Amst) 2003 2 11 1185 1197 10.1016/j.dnarep.2003.07.002 14599741

[B60] Lee AT Ren J Wong ET Ban KH Lee LA Lee CG The hepatitis B virus X protein sensitizes HepG2 cells to UV light-induced DNA damage J Biol Chem 2005 280 39 33525 33535 10.1074/jbc.M506628200 16055925

[B61] Kim CM Koike K Saito I Miyamura T Jay G HBx gene of hepatitis B virus induces liver cancer in transgenic mice Nature 1991 351 6324 317 320 10.1038/351317a0 2034275

[B62] Koike K Moriya K Yotsuyanagi H Iino S Kurokawa K Induction of cell cycle progression by hepatitis B virus HBx gene expression in quiescent mouse fibroblasts J Clin Invest 1994 94 1 44 49 10.1172/JCI117343 8040286 PMC296280

[B63] Slagle BL Lee TH Medina D Finegold MJ Butel JS Increased sensitivity to the hepatocarcinogen diethylnitrosamine in transgenic mice carrying the hepatitis B virus X gene Mol Carcinog 1996 15 4 261 269 10.1002/(SICI)1098-2744(199604)15:4<261::AID-MC3>3.0.CO;2-J 8634084

[B64] Terradillos O Billet O Renard CA Levy R Molina T Briand P Buendia MA The hepatitis B virus X gene potentiates c-myc-induced liver oncogenesis in transgenic mice Oncogene 1997 14 4 395 404 10.1038/sj.onc.1200850 9053836

[B65] Hoeijmakers JH Human nucleotide excision repair syndromes: molecular clues to unexpected intricacies Eur J Cancer 1994 30A 13 1912 1921 10.1016/0959-8049(94)00381-E 7734202

[B66] Chu G Mayne L Xeroderma pigmentosum, Cockayne syndrome and trichothiodystrophy: do the genes explain the diseases? Trends Genet 1996 12 5 187 192 10.1016/0168-9525(96)10021-4 8984734

[B67] Selby CP Sancar A Molecular mechanism of transcription-repair coupling Science 1993 260 5104 53 58 10.1126/science.8465200 8465200

[B68] Lindahl T Karran P Wood RD DNA excision repair pathways Curr Opin Genet Dev 1997 7 2 158 169 10.1016/S0959-437X(97)80124-4 9115419

[B69] Al-Mohanna MA Manogaran PS Al-Mukhalafi Z K AA-H Aboussekhra A The tumor suppressor p16(INK4a) gene is a regulator of apoptosis induced by ultraviolet light and cisplatin Oncogene 2004 23 1 201 212 10.1038/sj.onc.1206927 14712225

[B70] Goodrich JA Tjian R Transcription factors IIE and IIH and ATP hydrolysis direct promoter clearance by RNA polymerase II Cell 1994 77 1 145 156 10.1016/0092-8674(94)90242-9 8156590

[B71] Sancar A Excision repair in mammalian cells J Biol Chem 1995 270 27 15915 15918 7608140 10.1074/jbc.270.27.15915

[B72] Rossner MT Review: hepatitis B virus X-gene product: a promiscuous transcriptional activator J Med Virol 1992 36 2 101 117 10.1002/jmv.1890360207 1583465

[B73] Mathonnet G Lachance S Alaoui-Jamali M Drobetsky EA Expression of hepatitis B virus X oncoprotein inhibits transcription-coupled nucleotide excision repair in human cells Mutat Res 2004 554 1-2 305 318 10.1016/j.mrfmmm.2004.05.010 15450428

[B74] Tang H Oishi N Kaneko S Murakami S Molecular functions and biological roles of hepatitis B virus x protein Cancer Sci 2006 97 10 977 983 10.1111/j.1349-7006.2006.00299.x 16984372 PMC11159107

[B75] Ma NF Lau SH Hu L Xie D Wu J Yang J Wang Y Wu MC Fung J Bai X COOH-terminal truncated HBV X protein plays key role in hepatocarcinogenesis Clin Cancer Res 2008 14 16 5061 5068 10.1158/1078-0432.CCR-07-5082 18698024

[B76] Baptista M Kramvis A Kew MC High prevalence of 1762(T) 1764(A) mutations in the basic core promoter of hepatitis B virus isolated from black Africans with hepatocellular carcinoma compared with asymptomatic carriers Hepatology 1999 29 3 946 953 10.1002/hep.510290336 10051502

[B77] Kekule AS Lauer U Weiss L Luber B Hofschneider PH Hepatitis B virus transactivator HBx uses a tumour promoter signalling pathway Nature 1993 361 6414 742 745 10.1038/361742a0 8441471

[B78] Doria M Klein N Lucito R Schneider RJ The hepatitis B virus HBx protein is a dual specificity cytoplasmic activator of Ras and nuclear activator of transcription factors EMBO J 1995 14 19 4747 4757 7588604 10.1002/j.1460-2075.1995.tb00156.x PMC394572

[B79] Klein NP Schneider RJ Activation of Src family kinases by hepatitis B virus HBx protein and coupled signaling to Ras Mol Cell Biol 1997 17 11 6427 6436 9343405 10.1128/mcb.17.11.6427 PMC232495

[B80] Hsu T Moroy T Etiemble J Louise A Trepo C Tiollais P Buendia MA Activation of c-myc by woodchuck hepatitis virus insertion in hepatocellular carcinoma Cell 1988 55 4 627 635 10.1016/0092-8674(88)90221-8 3180223

[B81] Takada S Gotoh Y Hayashi S Yoshida M Koike K Structural rearrangement of integrated hepatitis B virus DNA as well as cellular flanking DNA is present in chronically infected hepatic tissues J Virol 1990 64 2 822 828 2296084 10.1128/jvi.64.2.822-828.1990 PMC249177

[B82] Buetow KH Sheffield VC Zhu M Zhou T Shen FM Hino O Smith M McMahon BJ Lanier AP London WT Low frequency of p53 mutations observed in a diverse collection of primary hepatocellular carcinomas Proc Natl Acad Sci USA 1992 89 20 9622 9626 10.1073/pnas.89.20.9622 1329103 PMC50184

[B83] Urano Y Watanabe K Lin CC Hino O Tamaoki T Interstitial chromosomal deletion within 4q11-q13 in a human hepatoma cell line Cancer Res 1991 51 5 1460 1464 1847664

[B84] Natoli G Avantaggiati ML Chirillo P Costanzo A Artini M Balsano C Levrero M Induction of the DNA-binding activity of c-jun/c-fos heterodimers by the hepatitis B virus transactivator pX Mol Cell Biol 1994 14 2 989 998 7507209 10.1128/mcb.14.2.989-998.1994 PMC358454

[B85] Natoli G Avantaggiati ML Chirillo P Puri PL Ianni A Balsano C Levrero M Ras- and Raf-dependent activation of c-jun transcriptional activity by the hepatitis B virus transactivator pX Oncogene 1994 9 10 2837 2843 8084589

[B86] Benn J Su F Doria M Schneider RJ Hepatitis B virus HBx protein induces transcription factor AP-1 by activation of extracellular signal-regulated and c-Jun N-terminal mitogen-activated protein kinases J Virol 1996 70 8 4978 4985 8764004 10.1128/jvi.70.8.4978-4985.1996 PMC190451

[B87] Huang SN Chisari FV Strong, sustained hepatocellular proliferation precedes hepatocarcinogenesis in hepatitis B surface antigen transgenic mice Hepatology 1995 21 3 620 626 7875658

[B88] Hsieh YH Su IJ Wang HC Chang WW Lei HY Lai MD Chang WT Huang W Pre-S mutant surface antigens in chronic hepatitis B virus infection induce oxidative stress and DNA damage Carcinogenesis 2004 25 10 2023 2032 10.1093/carcin/bgh207 15180947

[B89] Shinmura K Yokota J The OGG1 gene encodes a repair enzyme for oxidatively damaged DNA and is involved in human carcinogenesis Antioxid Redox Signal 2001 3 4 597 609 10.1089/15230860152542952 11554447

